# Automatic mining of symptom severity from psychiatric evaluation notes

**DOI:** 10.1002/mpr.1602

**Published:** 2017-12-22

**Authors:** George Karystianis, Alejo J. Nevado, Chi‐Hun Kim, Azad Dehghan, John A. Keane, Goran Nenadic

**Affiliations:** ^1^ Centre for Health Informatics Australian Institute of Health Innovation, Macquarie University Sydney Australia; ^2^ Department of Psychiatry University of Oxford Oxford UK; ^3^ The Christie NHS Foundation Trust Manchester UK; ^4^ School of Computer Science University of Manchester Manchester UK; ^5^ HerRC Health e‐Research Centre Manchester UK; ^6^ Faculty of Medicine The Kirby Institute, University of New South Wales Sydney Australia

**Keywords:** classification, neural networks, psychiatric evaluation records, rule‐based approach, text mining

## Abstract

**Objectives:**

As electronic mental health records become more widely available, several approaches have been suggested to automatically extract information from free‐text narrative aiming to support epidemiological research and clinical decision‐making. In this paper, we explore extraction of explicit mentions of symptom severity from initial psychiatric evaluation records. We use the data provided by the 2016 CEGS N‐GRID NLP shared task Track 2, which contains 541 records manually annotated for symptom severity according to the Research Domain Criteria.

**Methods:**

We designed and implemented 3 automatic methods: a knowledge‐driven approach relying on local lexicalized rules based on common syntactic patterns in text suggesting positive valence symptoms; a machine learning method using a neural network; and a hybrid approach combining the first 2 methods with a neural network.

**Results:**

The results on an unseen evaluation set of 216 psychiatric evaluation records showed a performance of 80.1% for the rule‐based method, 73.3% for the machine‐learning approach, and 72.0% for the hybrid one.

**Conclusions:**

Although more work is needed to improve the accuracy, the results are encouraging and indicate that automated text mining methods can be used to classify mental health symptom severity from free text psychiatric notes to support epidemiological and clinical research.

**Abbreviations**
ADE: adverse drug events
AI: artificial intelligence
AUDIT C‐SCORE: alcohol use disorders identification test—consumption score
CBT: cognitive behavioural therapy
DBT: dialectical behavioural therapy
DUI: driving under the influence
DWI: driving while intoxicated
EMR: electronic medical record
EMS: emergency medical services
ER: emergency room
ETOH: ethanol
GATE: General Architecture for Text Engineering
GS: gold standard
IOP: intensive outpatient program
**MAE**^**M**^: mean absolute error measure
MJ: marijuana
MMSE: mini mental state examination
N‐GRID: neuropsychiatric genome‐scale and RDoC individualized domains
OCD: obsessive compulsive disorder
OCSD: obsessive compulsive spectrum disorders
OUI: operating under the influence
PT: patient
RDoC: Research Domain Criteria
SMI: severe mental illness
TM: text mining
YBOCS: Yale‐Brown obsessive–compulsive scale

## 1 INTRODUCTION

Recent developments in the use and availability of electronic medical records (EMRs) have triggered a number of opportunities for more efficient clinical decision support and epidemiological research. EMRs contain information such as the patient's clinical history, treatments, and laboratory results (Abbe, Grouin, Zweigenbaum, & Falissard, 2015; Ford, Carroll, Smith, Scott, & Cassell, 2016; Thomas et al., 2014) and—when available on a larger scale—can provide a unique opportunity for clinical investigations, decision support, meta‐analysis, and observational research (Jackson et al., 2017; Kovalchuk, Stewart, Broadbent, Hubbard, & Dobson, 2017; Perlis et al., 2012). Specifically, psychiatric EMRs contain rich knowledge regarding the mental health status of patients and important contextual information that is often in free text. Unlike other disciplines, free text is a key means to record information in mental healthcare as there are few laboratory tests that can describe symptoms and their severity (unlike, e.g., measuring the blood pressure for hypertension). Even when specific instruments and tests (e.g., mini mental state examination) are used, they are most often reported in free‐text narrative. Mental healthcare therefore mainly relies on free text descriptions of symptoms, which are then interpreted, inspected, and assessed by health professionals in order to understand the type and the severity of the disease. A key question that we explore in this paper is whether we can automatically process such notes to extract disease severity for a given patient.

Processing of healthcare narrative has been a focus of clinical text mining and natural language processing for over 30 years, with notable results in automated harvesting of important clinical concepts and events in many domains (Abbe et al., 2015; Doan, Collier, Xu, Duy, & Phuong, 2012; Friedman, Shagina, Lussier, & Hripcsak, 2004; Savova et al., 2010; Sohn, Kocher, Chute, & Savova, 2011; Spasić, Livsey, Keane, & Nenadić, 2014). The main challenge is that clinical narrative is often written with a distinct style, seldom conforming to standard grammar, frequently with spelling and typing errors as well as common abbreviations and acronyms, with their meaning being often ambiguous depending on the context (Abbe et al., 2015; Dehghan, Keane, & Nenadic, 2013; Ford et al., 2016). Another challenge includes the extensive use of negations to rule out clinical signs and references to subjects other than the actual patient (Eriksson, Jensen, Frankild, Jensen, & Brunak, 2013).

Text mining has been also applied to free‐text data in the field of mental health. For example, Sohn et al. (2011) aimed to identify physician asserted drug side effects from psychiatric and psychological narratives through a hybrid approach of machine learning and rules, with an F‐score of 75%. Similarly, Eriksson et al. (2013) aimed to recognize possible adverse drug events from clinical narrative text in psychiatric hospital records with 89% precision through dictionaries and postcoordination rules in order to construct adverse drug events compound terms. Perlis et al. (2012) extracted outcomes of antidepressant treatments in major depressive disorders from EMRs through a supervised approach that involved logistic regression, with the precision ranging from 78% to 86%. Cunningham, Tablan, Roberts, and Bontcheva (2013) utilized a rule‐based approach for the extraction of mini mental state examination results from both short clinical notes and free text health record correspondence between clinicians with an overall precision ranging from 85% (in short notes) to 87% (in correspondence texts). Jackson et al. (2017) sought to capture a number of key symptoms of severe mental illness from clinical discharge summaries with a median F‐sore of 88% using regular expression pattern matching.

Several community challenges in clinical text processing have been organized to assess the state of the art for specific tasks, including comorbidity extraction (Uzuner, 2009), heart disease risk factors (Karystianis, Dehghan, Kovacevic, Keane, & Nenadic, 2015), and medication information (Uzuner, Solti, & Cadag, 2010). One of the tasks in the recent 2016 CEGS N‐GRID shared tasks focused on the determination of symptom severity for a patient based on information included in their initial psychiatric evaluation report (Filannino, Stubbs, & Uzuner, 2017). The classification regarding the severity of symptoms is important to understand if a patient requires immediate medical attention or hospitalization. In this paper, we describe and evaluate three approaches for the extraction of mental health symptom severity from psychiatric records in the context of the CEGS N‐GRID shared task. We have explored both knowledge‐driven (rule‐based) and data‐driven (ML based) methods to observe which one performed better for the given task. Specifically, the approaches include (a) a knowledge‐driven methodology based on lexicalized rules combined with manually constructed dictionaries characterizing positive valence symptoms; (b) a neural network (NN) built on lexical and semantic features extracted from the text; and c) a hybrid approach that combined the best predictions between the rule‐based and NN methods.

## 2 METHODS

### 2.1 Data and task

The data used contained 541 fully de‐identified initial psychiatric evaluation records provided by the Partners Healthcare and the Neuropsychiatric Genome‐Scale and Research Domain Criteria (RDoC) Individualized Domains (N‐GRID) project of the Harvard Medical School. The organizers released 325 psychiatric records that were used as the training set, and a set of 216 unseen psychiatric records for validation purposes. Each patient was represented by one record that included information from their initial psychiatric evaluation, containing unstructured free‐text narrative (e.g., “arrested for driving while intoxicated”) and structured question‐answer pairs (e.g., “History of Drug Use: Yes”). Each report (i.e., patient) has been classified with regard to the severity of experienced symptoms as: 0 (*absent* = no symptoms mentioned), 1 (*mild* = symptoms present but not a focus of treatment), 2 (*moderate* = a focus of treatment), and 3 (*severe* = requiring hospitalization or emergency room visit or equivalent). Through a thorough process, each report has been manually categorized in terms of symptom severity by two expert psychiatrists from the Massachusetts General Hospital and the Harvard Medical School; because psychiatrists may interpret the notes differently (which in this case happened in around 40% of cases), a third annotator adjudicated to generate the gold standard data (Filannino et al., 2017). The annotation process relied on the RDoC framework (Kozak & Cuthbert, 2016), which has been developed by the United States National Institute of Mental Health for the assessment of a patient's symptom severity in various domains. RDoC focuses on five psychiatric domains: (a) positive valence, (b) negative valence, (c) cognitive, (d) social processes, (e) arousal and regulatory systems—each characterized at different levels such as genomic, cellular, behavioural—depending on the type of available data. The CEGS N‐GRID Challenge focused only on mining psychiatric symptom severity that belonged to the positive valence domain: A domain that pertains to events and situations that the patient actively engages into, signalling mental health disorders ranging from behavioural ones (e.g., binge alcohol drinking and excessing marijuana use) to medical treatments (e.g., detoxification and inpatient treatment) and mental disorders (e.g., bulimia and mania). We note that we have used the CEGS N‐GRID gold standard data as provided, without further exploring or questioning the validity of the professional judgement of the severity classification provided in the annotated data sets.

**Figure 1:**
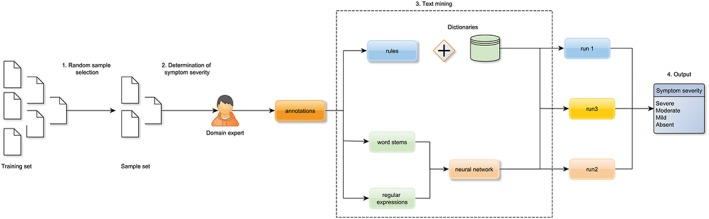
An overview of our hybrid approach for the determination of symptom severity in psychiatric evaluation records

The task we address here was to automatically extract severity for a given patient given their record. Figure 1 shows an overview of the approaches for the automatic classification of symptom severity: a rule‐based methodology (Section 2.2), a NN approach (Section 2.3), and a hybrid method (Section 2.4).

### 2.2 Rule‐based approach

#### 2.2.1 Dictionaries of symptoms

A sample of 50 records from the training data set randomly selected for each severity (13 for severe, 13 for moderate, 18 for mild, 6 for absent) was manually inspected by a domain expert (C. H. K.) to identify terms indicative of each severity level. The domain expert classified them into structured and unstructured mentions in text. In particular, structured mentions are represented by questions referring to particular symptoms followed by a “yes” or a “no” answer (e.g., “*History of Drug Use: Yes*,” “*Does the patient think they have an eating disorder: No*”). Any mention of a positive valence symptom that has been described in free text is labelled as unstructured (e.g., “*Mr. Andrade reported a chronic history of polysubstance dependence most notable opiate addiction”* [positive valence symptom]). Table 1 shows the symptoms identified for each class along with their classification as either structured or as unstructured mentions with a brief description and respective examples. We note that for the classes of severe and moderate severity, we did not take into consideration any structured questions as there were no discriminative questions.

**Table 1:** The list of the positive valence symptoms identified as relevant for the determination of symptom severity in initial psychiatric evaluation records

Class	Structured/ unstructured	Symptom	Description	Example(s)
Severe	Unstructured	Detox	Detoxification	Inpatient detox; 1st detox
		Arrest	Arrest for driving under the influence or intoxicated	Arrest for DUI; arrested for DWI
		Hospitalization	Psychiatric hospitalization	Requiring psychiatric hospitalization
		ER	Emergency room	Went to the ER; went to the emergency room
		IOP	Intensive outpatient program	Treatment included ×2 IOP treatment; IOP for alcohol dependence
		Liver transplantation	Liver transplantation due to substance abuse	Received liver transplant; liver transplantation
		Blackout	Blackouts due to substance abuse	Pt was having a daily blackout; had two blackouts
Moderate	Unstructured	Substance abuse	Abuse or dependence on various substances	303.90 alcohol dependence; history: History of alcohol abuse
		Treatment	Various psychological therapies/treatments	Would benefit from DBT; refer to CBT
		Mania	Suffering a manic episode	Having manic episodes; experienced a manic episode
		Eating disorder	Eating disorder diagnosis	307.5 eating disorder; diagnosed with bulimia
		Arrest	Arrest for driving under the influence or intoxicated	Arrest for DUI; arrested for DWI
		OCD	Obsessive compulsive disorder diagnosis	300.3 obsessive compulsive; Severe OCD
		YBOCS	Yale‐Brown obsessive compulsive scale	YBOSC‐sr 16; Ybosc: 20
		Naltrexone/Suboxone	Medication linked to mental health pharmacotherapies	Started on Suboxone; started Suboxone
Mild	Unstructured	Appetite	Decreased appetite	Poor appetite; low interest
		Motivation	Decreased motivation	Endorses anhedonia; lack of motivation
		Substance use	Substance use	Minimal MJ consumption; minimal etoh or mj use
	Structured	OCD	Obsessive compulsive disorder diagnosis	OCD: Does the patient struggle with repetitive unwanted thoughts or behaviors for at least 1 hr per day: Yes
		OCSD	Obsessive compulsive spectrum disorders positive mention	Obsessive compulsive spectrum disorders: Does the patient have other repetitive, unwanted thoughts or behaviors that are nonfunctional and difficult to stop (e.g., excessive preoccupation with appearance, hairpulling/skin picking, motor or vocal tics): Yes
		Depression	Depression diagnosis	Depression: Has the patient had periods of time lasting 2 weeks or longer in which, most of the day on most days, they felt little interest or pleasure in doing things, or they had to push themselves to do things: Yes
		Bipolar	Bipolar disorder diagnosis	Bipolar: Has patient ever had a period of time when he/she felt “up” or “high” without the use of substances: Yes
		Eating disorder	Eating disorder diagnosis	Eating disorders: Does the patient think they have an eating disorder: Yes
		Gambling behaviour	Positive gambling behaviour	Gambling behavior: Yes
		Drug use	Use of drugs	History of drug use: Yes
		Marijuana	Use of marijuana	Marijuana: Yes
		Cocaine	Use of cocaine	Cocaine: Yes
		Hypnotics	Use of hypnotics	Sedative‐hypnotics: Yes
		Stimulants	Use of stimulants	Stimulants: Yes
		Opiates	Use of opiates	Opiates: Yes
		Hallucinogens	Use of hallucinogens	Hallucinogens: Yes
		Prescription medication for non‐medical purposes	Use of other prescribed for nonmedical purposes medication	Prescription medications for nonmedical purposes: Yes
		Other substances	Use of other substances	Other substances: Yes
		Audit C‐score	Assessing a patient's alcohol consumption	Audit‐C total score: 5

*Note*. The symptoms are classified into structured or unstructured, depending on where they mainly appear. DUI = driving under the influence; DWI = driving while intoxicated; CBT = cognitive behavioural therapy; MJ = marijuana; DBT = dialectical behaviour therapy.

Following the initial inspection of the sample of records, a number of task‐specific semantic classes that represent various positive valence symptoms were manually organized into dictionaries with terms as well as potential abbreviations, synonyms, and acronyms (see Table 2, for some examples).

**Table 2:** Examples of manually assembled dictionaries, with their description and size

Dictionary name	Description	Examples	Size
Admission	Words suggesting hospitalization	Admits, hospitalization	6
DUI	Abbreviations for driving under the influence	DUI, OUI	3
Treatment	Therapies used to address mental health problems	Cognitive behavioural therapy, DBT	22
Abuse degree	Adjectives describing substance abuse	Severe, problematic	7
Abuse	Various types of substance abuse	Alcohol abuse, drug dependence	52
Substances	Substances linked with abuse	Etoh, cocaine	12
Mania	Terms describing manic episodes	Mania, hypomanic episode	6
Eating disorder	Terms describing various eating disorders	Bulimia, anorexia	9
Audit C‐score numbers	Numbers and words describing numbers related to the audit C‐score for medium risk of harm	5, seven	8
Neurovegetative symptoms	Symptoms related with depression	Sleep, energy	8
Motivation	Terms describing motivation and interest	Motivation, anhedonia	7

*Note*. DUI = driving under the influence; OUI = operating under the influence; DBT = dialectical behavioural therapy.

#### 2.2.2 Engineering information extraction rules

We created rules for each of the symptom severity classes for the recognition of mentions of positive valence symptoms. The rules are based on common lexical patterns identified in clinical notes (e.g., “been hospitalized for **eating disorder**” [symptom for moderate class]; “patient was arrested for driving under the influence [**DUI]**” [symptom for severe class]) that describe symptom mentions in text. The lexical patterns use (a) frozen lexical expressions as anchors for certain symptom mentions (e.g., “**parole:** history of driving while intoxicated,” “**Hallucinogens:** Yes”) based on verbs, noun phrases, and prepositions; and/or (b) semantic place holders (through dictionary mentions) suggesting the presence of a positive valence symptom (e.g., “history of substance abuse include **cocaine and alcohol consumption**,” “active in **AA**,” “leading to his **DUI**”). We have also implemented concept enumeration as it appears quite frequently in the training data, particularly for the reporting of various positive valence symptom mentions (e.g., “neurovegetative symptoms: **appetite [positive valence symptom]; interest [positive valence symptom]**”). Table 3 shows an example of a lexical pattern.

**Table 3:** An example of a lexical pattern to capture mentions of the severe class symptom of DUI arrest; “pt” is matched via a dictionary that contains variations of words representing “patient”; “was arrested for” is a semifrozen lexical expression for the identification of the positive valence symptom “DUI”

Lexical pattern	pt	[was] arrested for	DUI
	↓ Dictionary	↓ Semifrozen lexical expression	↓ Dictionary

*Note*. DUI = driving under the influence.

In order to create the rules, we used General Architecture for Text Engineering (Cunningham et al., 2013), a well‐established framework for text annotation and categorization. The observed lexical patterns in text were converted into rules using Java Annotations Pattern Engine, a pattern matching language for General Architecture for Text Engineering. Table 4 shows some rule examples, whereas Table 5 indicates the number of rules for specific symptoms roughly suggesting the complexity of the targeted information.

**Table 4:** Rule examples (using the GATE notation) for the recognition of positive valence symptoms for the severe class (e.g., matching “patient was having occasional blackouts,” aiming to identify any blackout episodes); moderate class (e.g., “her alcohol use has been problematic” aiming to identify the severity of the alcohol use); and mild class (“appetite is poor” aiming to identify low appetite)

Symptom							
Appetite	Example	**Appetite**		**Is**			**Poor**
	Rule	**{Token.string = ~”(?i)appetite”}**		**({Token.string = ~”(?i)is”}|{Token.String==“:”})**			**(volume)**
Substance abuse	Example	Her	**Alcohol**	**Use**	**Has**	**Been**	**Problematic**
	Rule	({Token.string = ~”(?i)her”}| {Token.string = ~”(?i)his”})	**(substances)**	**{Token.string = ~”(?i)use”}**	**({Token.string = ~”(?i)is”}| {Token.string = ~”(?i)has”})**	**({token})[0,1]**	**(degree)**
Blackout	Example	Patient	Was	Having	Occasional	**Blackouts**	
	Rule	({Token.string = ~”(?i)patient”}| {Token.string = ~”(?i)pt”})	{Token.string = ~”(?i)was”}	{Token.string = ~”(?i)having”}	({token})[0,1]	**{Token.string = ~”(?i)blackouts”}**	

*Note*. The extracted symptom mentions are highlighted in bold. The rules use strict token matching e.g., {Token.String = ~”(?I)appetite”} matches “appetite” (upper or lower case). Various dictionaries contain single and multiword terms (e.g., (volume), (degree), (substances) include words describing a decrease, the severity of the substance abuse, and the various substances that have been linked to abuse, respectively (see Table 2, for examples). {token})[0,1] matches any token. GATE = general architecture for text engineering.

**Table 5:** Number of rules engineered to represent each symptom severity class including unstructured symptom mentions and structured questions

Severity class	Rules for	Number of rules
Mild	Appetite	11
	Motivation	21
	Substance	5
	Miscellaneous	2
	OCD, OCSD, depression, bipolar, eating disorder, gambling behaviour, drug use, marijuana, cocaine, hypnotics, stimulants, opiates, hallucinogens, prescription medications for nonmedical purposes, other substances, C‐score	15*
Moderate	Degree abuse	33
	Treatment	16
	Eating disorder	21
	Mania	10
	Naltrexone/Suboxone	10
	YBOCS	1
	Arrest	6
	OCD	9
Severe	Detox	1
	Arrest for DUI	15
	Hospitalization	17
	ER	5
	IOP	3
	Liver transplantation	1
	Blackout	10

*Note*. Structured questions were used only for the mild class. The * in the mild class refers to the fact that each structured question for each symptom was represented by only one rule. OCD = obsessive compulsive disorder; OCSD = obsessive compulsive spectrum disorders; YBOCS = Yale‐Brown obsessive–compulsive scale; DUI = driving under the influence; ER = emergency room; IOP = intensive outpatient program.

More than one pattern may exist in a psychiatric note and might refer to one or more symptoms. Because we are aiming to classify each record according to the severance of its present symptoms, we combined the mention‐level classifications at the record level by considering precedence rules. For example, events with the highest clinical significance (i.e., close to severe) determined the rating of the whole patient document regardless of the number of lower severity events present (i.e., if a record has two symptoms belonging in different classes, this record will be assigned the highest in terms of severity class.).

### 2.3 Machine‐learning approach

In our machine learning approach, we implemented a NN approach because NNs are flexible for integrating different input data types into the same architecture, such as word counts, extracted values (e.g., age, gender, and other diagnoses), outputs coming from other text processing pipelines (e.g., rules), or even raw text in the form of word embeddings. Additionally, in the last 5 years, due to recent developments in hardware and software, NNs have become the state of the art in imaging, sound processing, and in certain areas of natural language processing (NLP; Goodfellow, Bengio, & Courville, 2016). Therefore, we decided to assess the utility of this technique in this task, which bears the added challenge of containing relatively few samples (in the order of hundreds) when compared to typical data sets used with NNs (in the order of millions).

The NN receives input from two sources that attempt extracting the most relevant information to the task from the original text. These two sources were a bag of word stems and a bag of strings matching a set of regular expressions. The bag of word stems was formed from 100 words regarded as highly relevant by a medical expert, plus the 100 stems most commonly occurring in the training set. The bag of regular expressions used a list of 52 simple expressions, aiming at capturing medically relevant events or symptoms. Table 6 shows the regular expressions used to capture mentions of addictive alcoholic behaviour. For the full list of the used regular expressions, see Table A1.

**Table 6:** Regular expressions used to detect mentions suggestive of addictive behaviour and alcoholic dependence

Regular expression	OR category
\bdependen	Addicted
\bdep\b	Addicted
\babuse\b	Addicted
\baddict	Addicted
\bwithdrawal sym	Addicted
\babstinen	Addicted
\bdrinking (?:often|daily|bing|daily|every|heav|prob|mis|over)	Alcoholic
\bpurging	Alcoholic
\bpurge\b	Alcoholic
\bAA\b	Alcoholic
Alcoholics anony	Alcoholic
\balcoholic	Alcoholic
\balcoholism	Alcoholic

*Note*. The regular expression to detect addiction was formed by the disjunctive combination (i.e., combination through the Boolean operation OR) of all regex with “OR category” addicted (indicated in second column). Similarly, the regular expression to detect alcohol dependence was formed by the disjunctive combination of all expressions in the alcoholic category. \b is used to represent the word boundaries.

**Figure 2:**
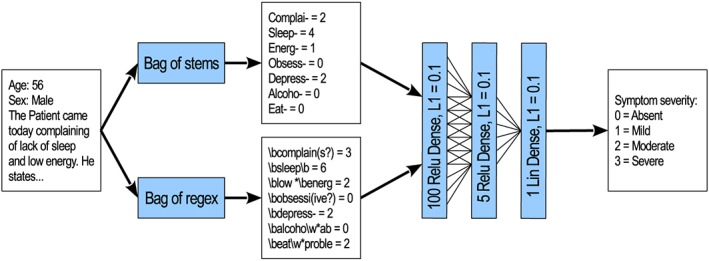
The neural network: From left to right: Depiction of an input text document; two input systems applying bag approaches to input document; bag results produced by each method for the input document; 3‐layered neural network; possible outputs of the neural network, treated as regression

The NN receiving the described inputs was formed by three densely connected layers, all of them using L1 regularization of 0.1. Although the first layer had 100 units with Relu transfer function, the second layer used 5 Relu units, and the last layer 1 linear unit. The output of the last layer was used as a regression of symptom severity, coded with values from 0 to 3 as indicated in Figure 2 below. The network was trained with backpropagation (stochastic gradient descent) on mean squared errors between the regressed output of the last layer and the real label of each sample. All code was implemented in Python with Keras library running over Theano, using 3 GTX 970 GPUs.

### 2.4 Hybrid approach

**Figure 3:**
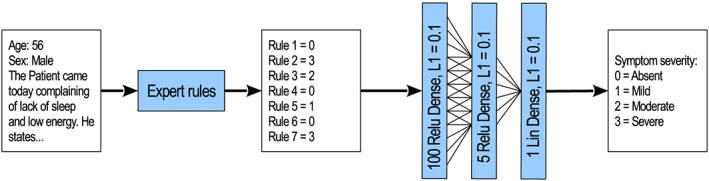
The neural network integration of the rule‐based approach. From left to right: Depiction of an input text document; input system based on rules; output of the input system, counting how many times each rule fired; 3‐layered neural network; possible outputs of the neural network, treated as regression

Finally, we combined the rule‐ and ML‐based approaches into a hybrid system. The hybrid system used the same architecture as the network described in Section 2.3, but used as an input the bag formed by the rules implemented in the rule‐based approach. Namely, the method counted how many times each rule fired (i.e., how many times the precondition of the given rule was true along the input document). The network was trained with backpropagation on mean squared error, as in Section 2.3. Figure 3 shows the architecture of the hybrid approach.

### 2.5 Evaluation metrics

The system has been formally evaluated as part of the 2016 N‐GRID challenge on a test‐set containing 216 unseen reports. In order to reduce the complexity of the N‐GRID task, only a single severity score per record was used, instead of having various scores for each positive valence symptom mention. As for the metric of evaluation, the macroaverage mean absolute error measure (MAE^M^) was used (Baccianella, Esuli, & Sebastiani, 2009). MAE^M^ measures the error and returns a percentage score (ranging from 0 to 100, with 100 being the highest performance). This metrics works well with imbalanced data by assigning the same importance to each severity class regardless of its frequency in the corpus by calculating how close the classification of each report was to the gold standard one. The following formula is used

$${\mathrm{MAE}}^{M}=\frac{1}{C}\;\sum_{j=1}^{C}\frac{1}{|D_{j}|}\;\sum_{x_{i}\in D_{j}}|h\left(x_{i}\right)-y_{i}|,$$

where C is the number of classes of interest (4 in this case); D_j_ is a set of records with score j; x_i_ is a record; h(x_i_) represents the predicted score with y_i_ being the correct one.

## 3 RESULTS

Table 7 displays the performance on the training and evaluation sets for all the approaches. On the evaluation set, the rule‐based method achieved the highest performance with 80.1%, whereas the NN method achieved 73.4%. The hybrid approach returned the lowest score (72.1%). Despite the drop in the performance compared to the training data, the rules generalized better than the data‐driven approaches.

**Table 7:** The performance (the MAE^M^ scores) for the classification of symptom severity for the training and evaluation sets for each class and applied method; NN refers to the neural network; hybrid is the combination of rules with the neural network; # is the number of examples in each data set

Class	Training set				Evaluation set			
	#	Rules	NN	Hybrid	#	Rules	NN	Hybrid
Absent	45	92.6	98.0	96.1	53	81.7	92.4	95.7
Mild	130	88.4	95.2	91.8	46	92.4	72.6	84.8
Moderate	82	84.7	92.1	89.2	86	69.5	67.4	54.3
Severe	68	94.1	87.3	81.4	31	76.7	61.0	53.5
MAE^M^ score		89.9	93.0	89.3		80.1	73.4	72.1
Total number of documents	325				216			

*Note*. MAE^M^ = mean absolute error measure.

Table 8 shows a confusion matrix with the numbers of reports that were correctly and incorrectly classified by the rule‐based approach in the evaluation set. By far, the biggest confusion was among mild and moderate cases (76 mild cases predicted as moderate, and 20 moderate cases predicted as mild), with some confusion between severe and moderate reports. Still, we note that there was not much confusion between extreme cases (i.e., severe and mild), which is reflected in relatively high MAE^M^ scores.

**Table 8:** Confusion matrix for the rule‐based approach that shows the number of documents classified in each class by the rules (vertically) and by the gold standard (horizontally)

Gold standard	Rules				
	Severe	Moderate	Mild	Absent	Total number of documents
Severe	27	16	9	1	53
Moderate	4	20	20	2	46
Mild	3	5	76	2	86
Absent	2	1	9	19	31
Total number of documents	36	42	114	24	216

*Note*. For example, 4 moderate cases where predicted as severe; on the other hand, 16 severe cases where misclassified as moderate.

## 4 DISCUSSION

### 4.1 Rule‐based approach

While inspecting the training set sample, we noticed that symptom severity could be assessed more accurately (and consistently) by observing the relevant unstructured text. For example, despite having positively answered some structured questions indicating severe and moderate severity, the overall record would still not be classified as severe or moderate. For example, certain structured questions indicating the severe class could be present in reports that were classified as moderate or mild (e.g., “Psychiatric History Hx of Inpatient Treatment: Yes”). Consequently, we chose to base our rules for all severity classes on unstructured symptom mentions in order to avoid the generation of false positives. Still, we included processing of structured questions in the mild class aiming to avoid overclassification of the remaining records as absent (because the frequency of unstructured mild class symptom mentions was quite small).

We performed manual examination of the errors in the evaluation data set with 74 records assigned the wrong symptom severity; 24 records were assigned a higher class than the correct one; and 50 records were assigned a lower severity class.

We note that in more than half of cases where a higher class was assigned, the reports mention symptoms that could indicate such higher severity. For example, correct recognition of a moderate symptom (e.g., “**304.03 Opioid dependence**,” “**History of drug use: Yes**”) in the unstructured part does not necessarily agree with the classification provided at the gold standard (which was mild in the above cases). Also in a quarter of cases, the rules did not deal with negated context (e.g., “parole: **no operating under the influence/DUI**,” “no **h/o detox**,” “She **denies** any difficult with sleep, appetite, energy”). There were few cases associated with symptoms of family members that were wrongly associated with the patient (e.g., **“grandmother hospitalized after** suicide attempt”). Finally, another source of errors were treatment plans that were not identified as such (e.g., “Will initiate an outpatient **detox** with long‐acting …”).

The cases where lower severity was wrongly assigned are mainly due to the fact that we have focused on capturing unstructured symptom mentions with the exception of structured mentions of the mild class. This choice led to several falsely classified documents to less severe classes. For example, four records had a structured question suggesting a symptom but we have decided that it was best for our system's performance to focus on unstructured symptom mentions only. Additionally, an unstructured mention of “**Alcohol abuse**” (indicating moderate severity) was overwritten as mild severity by identifying the structured mention of “**Marijuana: Yes**”). In five records, mentions of specific drugs indicated a certain severity level, but were not included in our rules (e.g., “Trial of **ritalin [symptom for the moderate class]**”). In a similar fashion, another source of errors (almost a third) was due to expression patterns that have not been spotted in the training data (e.g., “**residential treatment**” for the severe class, “**trichotillomania**” for the moderate class).

### 4.2 Neural network and hybrid approaches

The data‐driven approaches exemplify what is, as of now, the biggest challenge for deep NNs—namely, the dependence on having to use a large prelabelled corpus. Although deep NNs are becoming the state of the art in NLP when enough annotated text exists (Hashimoto, Xiong, Tsuruoka, & Socher, 2016), this is proving more difficult in tasks where labelled data is scarce. Other traditional artificial intelligence techniques, such as rules, are able to complement the lack of data by incorporating large amounts of expert knowledge, and this is what our rule approach has successfully achieved. However, the idea of incorporating expert knowledge into NNs is proving to be more challenging.

The biggest achievement on incorporating prior NLP knowledge into a NN has come in the form of word embeddings (such as Word2vec [Mikolov, Chen, Corrado, & Dean, 2013] and Glove [Pennington, Socher, & Manning, 2014]), which have dramatically improved the performance of deep learning in NLP tasks. Other attempts of incorporating potentially more abstract preknowledge into NNs, include pretraining the network in a larger annotated corpus, and then using the weights obtained in the first layers as initial values of the backpropagation when training in the final task of interest (Mou et al., 2016). However, the increase in performance of this second approach falls short of the improvement that was once achieved when first introducing embeddings into NNs, or with the use of expert knowledge in more traditional artificial intelligence approaches. The lower performance in our two NN approaches (including the hybrid system), exemplifies the difficulty that introducing expert knowledge into NNs poses. Although the expert rules used in our first method perform well by themselves, their accuracy notably drops when the NN attempts at learning to use these rules by applying on the training data. Not even the application of cross‐validation on the training data prevented the network from overfitting the evaluation set.

### 4.3 Limitations and future work

We designed our rules after exploring a rather small sample of the training set (50 records). Perhaps, a larger set might have helped engineering more rules that would able to cast a wider net to identify mentions of positive valence symptoms in text. Our emphasis on unstructured symptom mentions might also be a limitation as information from structured questions is disregarded instead of being taken into consideration for the classification of a record (with the exception of the mild class). Initially, we considered structured questions for all classes but we noted that they were not discriminative and would produce a number of errors. Perhaps, they can be useful in cases where there is not a single unstructured mention that can help for the recognition of the class severity.

Our precedence rules might be another limitation. Choosing the highest severity symptom to characterize (i.e., classify) a record (and ignoring mentions of other symptoms of lower severity) has led to some misclassifications. Combining different severity signals could be an interesting task to explore in the future.

All the methods developed here have been trained on initial psychiatric evaluation records. Although both rules and the data‐driven approaches may work on reports from other organizations, it is likely that they would need adjustments both in lexical and expression coverage. However, the rules and language models developed here focus on severity of symptom expressions and therefore may be used to process other types of mental health records.

## 5 CONCLUSION

We have designed and explored three methods of the identification of positive valence symptom severity in initial psychiatric evaluation records. The performance is promising, ranging from 72% (hybrid) to 80% (rule‐based). We noted that the lexicalized rules managed to generalize automatic classification of symptom severity relatively well. However, NNs, at least as implemented in this study, failed to generalize in the final evaluation set, even when 10‐fold cross‐validation results with the training set suggested that no severe overfitting was being produced. Combining the NN with rules in a hybrid serial system did not ameliorate the lack of generalization. Although there is still significant room for improvement, the results are encouraging and indicate that automated text mining methods can be used to classify mental symptom severity from psychiatric notes with a range of reasonably promising performance.

## DECLARATION OF INTEREST STATEMENT

The authors have no conflicts of interest to declare.

## APPENDIX 1

### 1.1

**Table A1:** The complete list of the used regular expressions for the detection of positive valence symptoms mentions. \b Is used in regular expressions to represent the word boundaries.

Regular expression	OR category
\bdependen	Addicted
\bdep\b	Addicted
\babuse\b	Addicted
\baddict	Addicted
\bwithdrawal sym	Addicted
\babstinen	Addicted
\bdrinking (?:often|daily|bing|daily|every|heav|prob|mis|over)	Alcoholic
\bpurging	Alcoholic
\bpurge\b	Alcoholic
\bAA\b	Alcoholic
Alcoholics anony	Alcoholic
\balcoholic	Alcoholic
\balcoholism	Alcoholic
Compulsive	Ocd
\banxious	Anxious
\banxiety	Anxious
\bfear	Anxious
\bpanic	Anxious
\brumina	Anxious
\bshy\b	Anxious
\bphobia	Anxious
GAD	Anxious
Xanax	Anxious
\bpalpitation	Anxious
Alprazolam	Anxious
Stress	Anxious
\bdespair	Anxious
\barrested\b	Arrested
\bcharged with	Arrested
\bcharges\b	Arrested
\brestraining order	Arrested
Legal history	Arrested
Bipolar	Bipolar
Eating disorders	Bulimia
\bCBT\b	Therapy
Cognitive behavioural	Therapy
\bHallucinat	Demented
Dementia	Demented
Brain injury	Demented
Migraine	Demented
Headache	Demented
Grief	Depressed
Depression	Depressed
\detox\b	Detox
Hx of inpatient	Detox
\bsteroid	Drug
Sedative‐hypnotics	Drug
Stimulants	Drug
Medications for non‐medical	Drug
\bER\b	Er
\bEMR\b	Er
\bEMT\b	Er
Emergency room	Er
Gambling behavior	Gambling
\bhospitali	Hospital
\admitted	Hospital
\badmission	Hospital
\admit\b	Hospital
\bdischarg	Hospital
\bresidential	Hospital
\bIOP\b	Iop
Intensive outpati	Iop
\bliver op	Liver
\bliver tra	Liver
\bluvox	Medication
\bSertraline	Medication
\bZoloft	Medication
\bcitalopram	Medication
\bLexapro	Medication
\bescitalopram	Medication
\bLorazepam	Medication
\bClonazepam	Medication
\bcelexa	Medication
\bklonopin	Medication
\bmedication\b	Medication
\bFluvoxamine	Medication
\bcompuls	Ocd
\bobsession	Ocd
OCD	Ocd
\btics?\b	Ocd
Obsessive compulsive	Ocd
PTSD	PTSD
Traumatic stre	PTSD
\bSIB\b	Sib
NSSI	Sib
\bself injury	Sib
Hx of non suicidal self injurious	Sib
Other substances	drugAdicted
\bkill himself	Suicide
\bkilling himself	Suicide
\bSA\b	Suicide
\bsuicid	Suicide
Hx of suicidal behavior	Suicide
Audit‐c	Test
Audit c	Test
YBOCS	Test
Yale Brown obsessive compulsive	Test
Alcohol use disorders identification	Test
\btherapy\b	Therapy
Cognitive behavioral therapy	Treatement
Dialectical behavioral therapy	Treatement
Addiction treatment	Treatement
Medication treatment	Treatement
Substance use intervention action plan	Treatement
Very angry	Violent
\bAggressiv	Violent
Hx of violent behavior	Violent
\bvodka	Alcoholic
\bwhiskey	Alcoholic
\bbeer	Alcoholic
\bETOH\b	Alcoholic
\balcohol	Alcoholic
\bdrinking\b	Alcoholic
\bAUD\b	Alcoholic
\bless motivat	Anhedonia
\bdemotivat	Anhedonia
\bamotivat	Anhedonia
\banhedon	Anhedonia
Interest	Anhedonia
Initiativ	Anhedonia
Pleas	Anhedonia
Libido	Anhedonia
\beat	Appetite
Appetite	Appetite
\bbipolar	Bipolar
\bBPAD\b	Bipolar
\bED\b	Bulimia
\beating disorder	Bulimia
\beating problem	Bulimia
\beating dif	Bulimia
\bbulimia	Bulimia
\bbinge\b	Bulimia
\bBED\b	Bulimia
Other substances	Drug
Hallucinogens	Drug
Cocaine	Drug
Opiates	Drug
Hypnotics	Drug
Poly? Substance	Drug
\bpolysub\b	Drug
\bIVDU\b	Drug
Intravenous drug	Drug
\bcannabis\b	Drug
\bcanabis\b	Drug
\bmarijuana\b	Drug
\bMJ\b	Drug
\bopiate	Drug
\bopioid	Drug
\bhallucinogen	Drug
\bmethamphetamine	Drug
\bamphetamine	Drug
\billicit drug	Drug
\bilegal drug	Drug
\billicit substance	Drug
\bilegal substance	Drug
\bgambling	Gambling
\bgamble	Gambling
\bhypomania	Manic
\bpsychosis	Manic
\bmania	Manic
\bmanic\b	Manic
Increas(?:e|ed|ing)? Spending	Manic
Increas(?:e|ed|ing)? Sex	Manic
\bOCD\b	Ocd
Obsessive compulsive	Ocd
Substance abuse	Drug
Substances? Related disorders?	Drug
\bsubstances? Use	Drug
Alcohol abus	Alcohol
\bunemploy	Work
SSDI	Work
Health	Health
\bmotivat	Motivation
\bcousin	Family
\bfamily\b	Family
\bsister	Family
\bbrother	Family
\bmother\b	Family
\bfather\b	Family
\bsibling	Family
\bAverage	Normal
Appropriate	Normal
\bnormal	Normal
Independent	Normal
WNL	Normal
\boveruse	Abuse
\babuse	Abuse
\bawful	Bad
\bbad	Bad
\bsevere	Bad
\bdown	Bad
\bpoor	Bad
\brumiat	Rumiation
